# A Streamlined Workflow for Microscopy-Driven MALDI Imaging Mass Spectrometry Data Collection

**DOI:** 10.1021/jasms.4c00365

**Published:** 2024-11-07

**Authors:** Allison B. Esselman, Megan S. Ward, Cody R. Marshall, Ellie L. Pingry, Martin Dufresne, Melissa A. Farrow, Matthew Schrag, Jeffrey M. Spraggins

**Affiliations:** † Mass Spectrometry Research Center, Vanderbilt University, Nashville, Tennessee 37240, United States; ‡ Department of Chemistry, Vanderbilt University, Nashville, Tennessee 37240, United States; § Chemical and Physical Biology Program, Vanderbilt University School of Medicine, Nashville, Tennessee 37232, United States; ∥ Department of Cell and Developmental Biology, Vanderbilt University, Nashville, Tennessee 37232, United States; ⊥ Department of Biochemistry, Vanderbilt University, Nashville, Tennessee 37232, United States; # Department of Neurology, Vanderbilt University School of Medicine, Nashville, Tennessee 37232, United States; 7 The Vanderbilt Brain Institute, Vanderbilt University, Nashville, Tennessee 37240, United States; 8 Vanderbilt Memory and Alzheimer’s Center, Vanderbilt University Medical Center, Nashville, Tennessee 37232, United States; 9 Department of Pathology, Microbiology, and Immunology, Vanderbilt University Medical Center, Nashville, Tennessee 37232, United States

**Keywords:** multimodal, molecular imaging, high spatial resolution imaging, human brain, HeLa cells, human kidney, high-throughput, targeted, targeted sampling, single-cell analysis

## Abstract

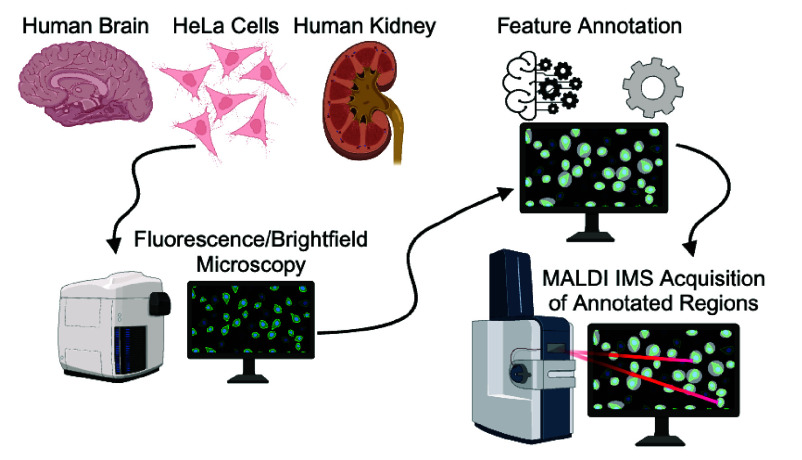

Matrix-assisted laser desorption/ionization imaging mass spectrometry (MALDI IMS) is a rapidly advancing technology for biomedical research. As spatial resolution increases, however, so do acquisition time, file size, and experimental cost, which increases the need to perform precise sampling of targeted tissue regions to optimize the biological information gleaned from an experiment and minimize wasted resources. The ability to define instrument measurement regions based on key tissue features and automatically sample these specific regions of interest (ROIs) addresses this challenge. Herein, we demonstrate a workflow using standard software that allows for direct sampling of microscopy-defined regions by MALDI IMS. Three case studies are included, highlighting different methods for defining features from common sample types—manual annotation of vasculature in human brain tissue, automated segmentation of renal functional tissue units across whole slide images using custom segmentation algorithms, and automated segmentation of dispersed HeLa cells using open-source software. Each case minimizes data acquisition from unnecessary sample regions and dramatically increases throughput while uncovering molecular heterogeneity within targeted ROIs. This workflow provides an approachable method for spatially targeted MALDI IMS driven by microscopy as part of multimodal molecular imaging studies.

## Introduction

Matrix-assisted laser desorption/ionization (MALDI) imaging mass spectrometry (IMS) is a powerful technology for mapping the localization of molecules across cells and tissues. Instrument advancements and sample preparation optimizations have enabled routine high spatial resolution MALDI imaging (≤10 μm pixel size).^1,2^ Often, these experiments are bolstered by microscopy measurements as part of multimodal studies to more directly connect IMS-based molecular measurements to critical cell types and histopathological features.^3,4^ A common challenge for IMS studies is defining specific measurement regions that target these key regions of interest (ROIs) while excluding unimportant sample areas or off-sample regions of the sample substrate.

Complementary microscopy can be used for ROI selection to drive IMS data acquisition of targeted sample features. This has been shown to dramatically improve throughput and reduce the total number of pixels collected, data file sizes, and experiment cost, which is particularly important for high spatial resolution IMS experiments. Measurement regions are often defined manually by “drawing” ROIs within instrument software, a burdensome and error-prone process for large-scale studies. To address this, we have previously demonstrated an autofluorescence microscopy-driven IMS method for targeting hundreds of glomeruli in human kidney tissue.^5,6^ However, these previous studies required complex, in-house scripts to convert glomeruli segmentation maps into measurement regions that could be utilized by instrument operation software to drive sampling.

Here, we demonstrate a streamlined workflow for targeting sample features for high spatial resolution MALDI IMS, making microscopy-driven multimodal IMS experiments more accessible to the broader research community. The case studies provided demonstrate the utility of this method for both tissue imaging and dispersed single-cell experiments, as well as the versatility in the types of segmentation strategies that can be implemented.

## Experimental Section

Alzheimer’s disease (AD)- and cerebral amyloid angiopathy (CAA)-impacted human brain and normal human kidney tissues were sectioned and mounted onto ITO-coated glass slides, and HeLa cells were grown directly on ITO-coated glass slides. Autofluorescence microscopy was acquired before IMS sample preparation. Samples were washed with ammonium formate, and 4-(dimethylamino)cinnamic acid (DMACA) was sublimated onto the slides.

ROIs were segmented based on autofluorescence or brightfield images for each sample. Segmentation of vasculature regions in the human brain was done with the wand and brush tools in QuPath, while HeLa cells were detected and segmented automatically with the Cell Detection feature in QuPath.^7^ Segmentation of glomeruli in human kidney with a 1.4× expanded border was performed using a previously described method with an in-house developed machine-learning model.^5,8,9^ All annotation files were saved as *.geojson* files.

Experiments were performed on a timsTOF fleX mass spectrometer with a microGRID stage (Bruker Daltonics, Bremen, Germany) in negative ion mode with a 5 μm stage raster.^1^ The autofluorescence image of each tissue section was aligned to the sample carrier position, a process referred to as “teaching”. The GeoJSON annotation files were imported into flexImaging using the import regions feature to automatically define predetermined ROIs for data acquisition. This new software feature is available in flexImaging v7.5. Figure S1 summarizes this workflow, and additional experimental information is provided in Methods S1. All ion images and spectra were visualized using SCiLS Lab (2024b).

## Results and Discussion

**Figure 1:**
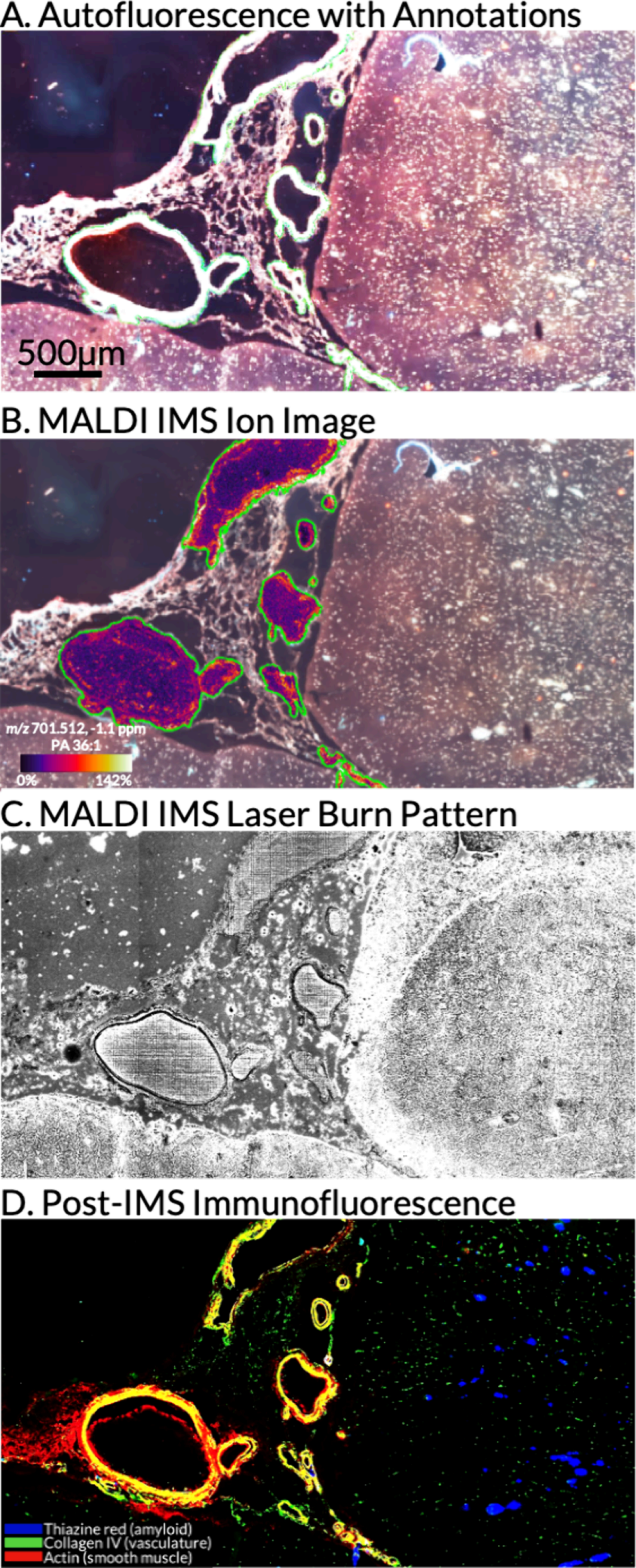
Targeted multimodal imaging of vasculature in the frontal cortex of the human brain. The autofluorescence image of the tissue section was used to manually annotate 17 vasculature ROIs in the tissue section (A). A MALDI IMS ion image of vasculature ROIs overlaid onto the autofluorescence image demonstrates the heterogeneity of PA (36:1) (*m*/*z* 701.512, −1.1 ppm) within the vasculature regions (B). The post-MALDI IMS brightfield microscopy of the laser ablation craters (C), and post-MALDI IMS IF stain show the specific targeting of the vasculature with this MALDI IMS experiment (D). In the IF image, amyloid is shown in blue (thiazine red), vasculature is shown in green (collagen IV), and smooth muscle is shown in red (actin).

This workflow allows segmentations to be determined by any means, manually or automatically, to define instrument measurement regions using the GeoJSON file format.^10^ The GeoJSON file format is a common open standard format that encodes spatial features (e.g., points and polygons) using JavaScript object notation, making this workflow agnostic to how software segmentations are generated. For example, QuPath,^7^ an open-source digital pathology software, was used to manually annotate cerebral vasculature based on autofluorescence intensity (Figures 1A and S2). In total, 17 segmented ROIs were imported into the instrument control software for targeted MALDI IMS data acquisition. Figure 1B highlights an ion image from this experiment showing an example ion distribution of a phosphatidic acid (PA) lipid localized to vasculature regions. To validate that these tissue structures were targeted, a post-IMS microscopy image collected from the tissue with the matrix remaining shows the ablation craters from the MALDI IMS experiment in the selected regions (Figure 1C), and these ablation craters can be aligned to vasculature features through coregistered immunofluorescence (IF) microscopy data from the same tissue section after matrix removal (Figure 1D). Targeting vasculature in human brain tissue is important in the study of the molecular mechanisms of CAA, one of several AD-associated pathologies. CAA is characterized by toxic β-amyloid deposits within cerebral and meningeal vessels and is challenging to study due to the sparsity and shape irregularity of vasculature tissue.^11−14^ Performing manual annotations this way allows imaging regions to be preselected by expert pathologists to support histology-targeted sampling of these critical neuropathological tissue features. Also, precisely selecting vasculature regions reduced the total number of pixels collected from 1,711,697 to 73,181 when compared to a rectangular region covering the same area, a reduction of 95% in time and instrument resources.

**Figure 2:**
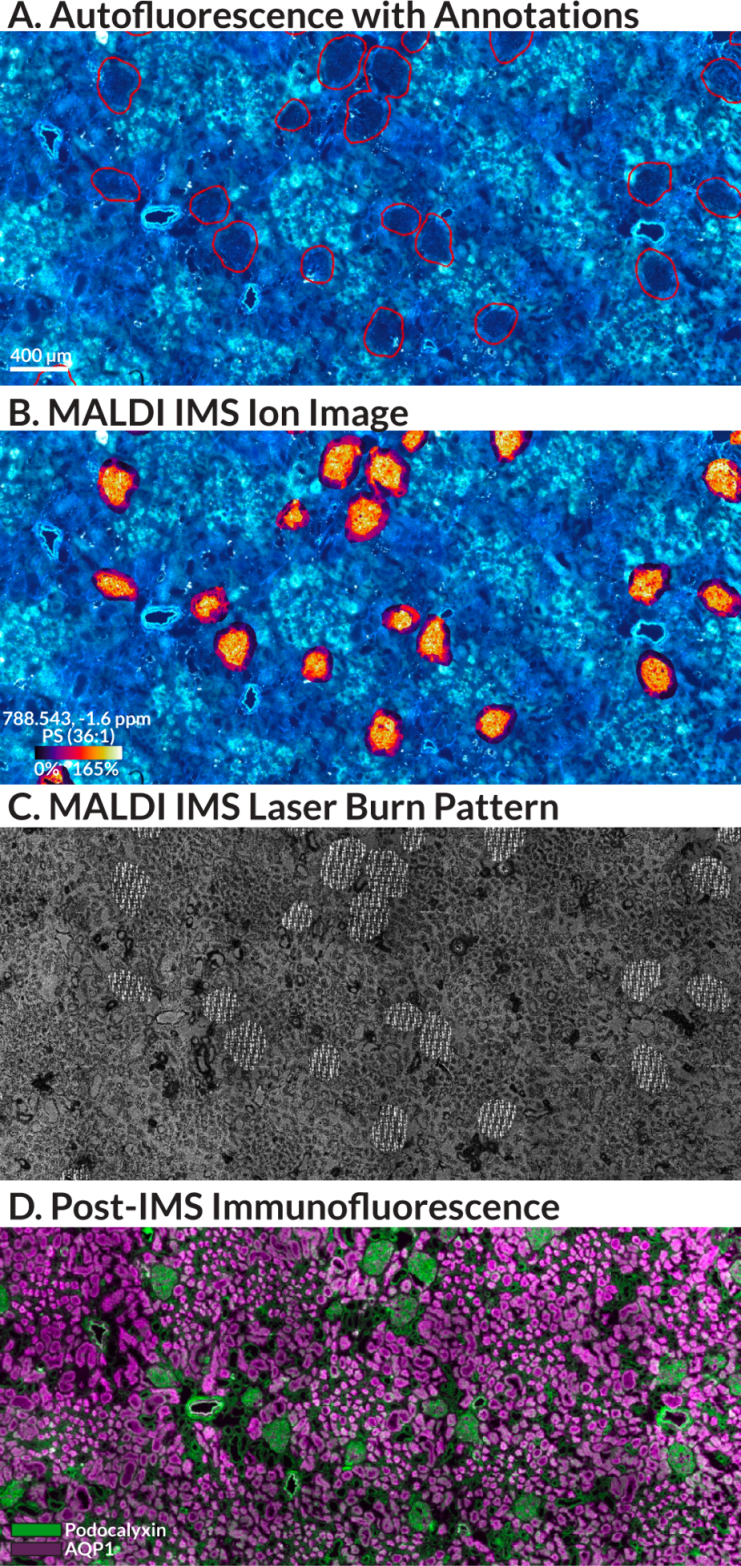
Specific targeting of glomeruli in human kidney. The autofluorescence image of the tissue section was used with a custom segmentation model to annotate 304 glomeruli in the tissue section (A). A MALDI IMS ion image of glomeruli overlaid onto the autofluorescence image shows the localization of PS (36:1) (*m*/*z* 788.543, −1.6 ppm) specifically to glomeruli (B). The post-MALDI IMS acquisition laser burn pattern (C), and post-MALDI IMS IF stain show the specific targeting of glomeruli in the tissue section. In the IF image, glomeruli are shown in green (podocalyxin), and proximal tubules are shown in pink (aquaporin 1) (D).

This workflow can also leverage automated machine-learning-derived segmentation masks to define MALDI IMS measurement regions. For example, autofluorescence and IF microscopy can be used to distinguish complex biological structures, such as the unique functional tissue units of the nephron. We have developed custom models to annotate glomeruli, proximal tubules, the loop of Henle, distal tubules, and collecting ducts in human kidney tissue based on autofluorescence microscopy to enable multimodal studies and unique IMS data mining strategies.^9^ For this case study, we segmented 304 glomeruli, the primary filtration unit of the kidney, from a single tissue section using the autofluorescence image as shown in Figures 2A and S3.^5,8,9^ These segments were then used as measurement regions for MALDI IMS providing molecular maps for all glomeruli across this whole slide image. This approach reduces the total number of pixels by 89% compared to collecting IMS data from the entire tissue section, and using automated segmentation algorithms allows high numbers of measurement regions to be defined and imported in minutes compared to the hours it would take to perform this task manually. Figure 2B shows an example ion image of a phosphatidylserine (PS) lipid localizing to glomeruli. As in the previous case study, brightfield microscopy was used to visualize the laser ablation craters (Figure 2C), and post-MALDI IF microscopy (Figure 2D) validated that the glomeruli were specifically sampled using this approach. The ability to automatically target hundreds of complex tissue structures and specific cell types across whole slide images is key for performing high spatial resolution MALDI imaging as part of large-scale studies. Glomeruli are just one example of such tissue features. Whether studying pancreatic islets in diabetic patients or amyloid plaques in AD, it is becoming increasingly necessary to generate high spatial resolution MALDI imaging data across vast tissue areas to fully elucidate localized molecular events associated with disease. Targeted workflows are necessary for making these experiments practical.

**Figure 3:**
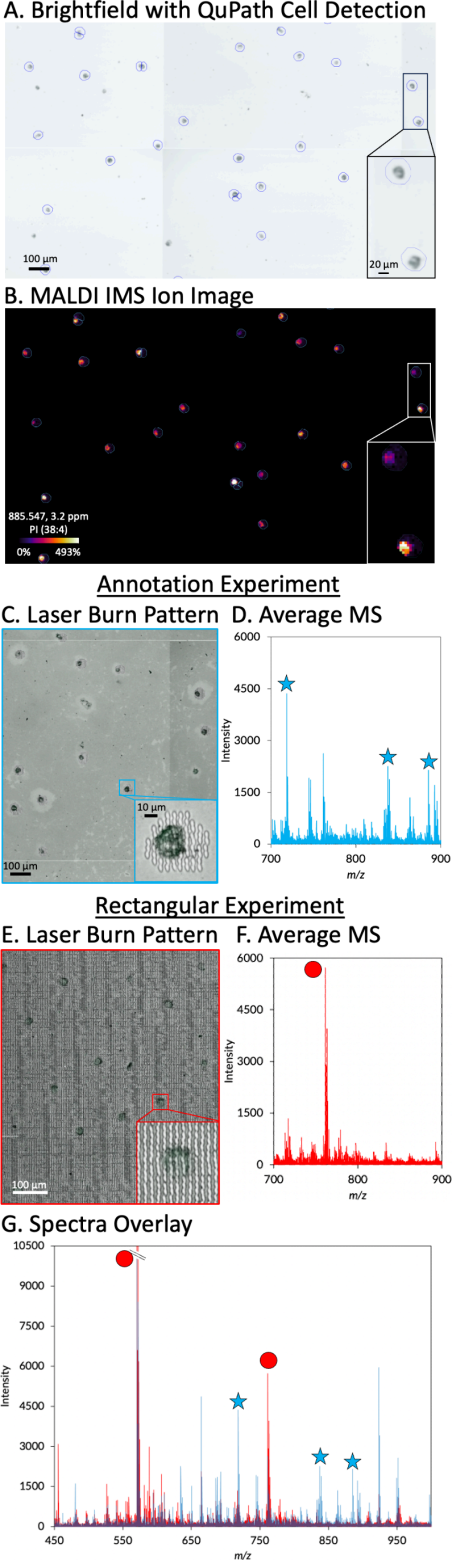
Targeted acquisition of HeLa cells. The QuPath Cell Detection feature was used on a brightfield image to define approximately 1000 ROIs (A). A MALDI IMS ion image of PI (38:4) (*m*/*z* 885.547, 3.2 ppm) localized to only the HeLa cells (B). Laser ablation craters from the targeted MALDI IMS experiment (C), and the average mass spectrum from those regions from *m*/*z* 700–900 with peaks noted with a blue star tentatively identified based on accurate mass as PE (34:0) (*m*/*z* 718.538), PI (34:0) (*m*/*z* 837.547), and PI (38:4) (*m*/*z* 885.547) (D). Laser ablation craters from the rectangular MALDI IMS experiment (E), and the average mass spectrum from the region displayed from *m*/*z* 700–900 where the peak noted with a red circle is a matrix peak with an *m*/*z* of 761.354 (F). Average mass spectra from the targeted experiment (blue) overlaid with the rectangular region experiment (red) show decreased matrix and noise peaks in the targeted experiment compared to the rectangular region (G). The red circles indicate matrix or nonbiological peaks, whereas the blue stars indicate biologically significant peaks. For example, the peaks with red circles are matrix peaks with an *m*/*z* of 570.260 and 761.354, respectively. The peaks with blue stars are tentatively identified based on accurate mass as PE (34:0) (*m*/*z* 718.538), PI (34:0) (*m*/*z* 837.547), and PI (38:4) (*m*/*z* 885.547). The intensity of the condensed peak G is 17,290.

Beyond tissue imaging, analyzing dispersed cells is an emerging approach to assess single-cell biology using MALDI IMS.^15,16^ These experiments present a unique challenge as imaging regions often contain significantly more noncellular pixels, unnecessarily increasing acquisition time, file size, and chemical noise. Methods have been developed using custom, specialized approaches for targeting cells, making this an ideal case study to demonstrate our refined workflows.^17^ Here, approximately 1,000 HeLa cells were annotated based on microscopy using QuPath’s Cell Detection tool.^7^ The annotations were imported into the instrument data acquisition software as individual ROIs for MALDI IMS analysis (Figures 3A and S4). Figure 3B shows an example ion image of a phosphatidylinositol (PI) lipid from the imaged HeLa cell regions. For comparison, MALDI IMS data was collected from a rectangular region containing cells and matrix. Figure 3C displays a section of the burn pattern from the targeted experiment and the average mass spectrum from that area (Figure 3D). Comparatively, the burn pattern and average mass spectrum from the rectangular region experiment are shown in Figures 3E and 3F, respectively. The number of pixels containing cellular information in the rectangular measurement region was only 2.1% of the total ROI, as determined by the measurement region shown in Figure 3E. This leads to a dramatic reduction in the required time of the experiment. Here, IMS data were generated from 999 cells in just 90 min. It is noted that for cases where measurement regions are relatively small compared to the overall sample area, like this example and the previous case study assessing glomeruli, special care must be taken to ensure that microscopy images used for segmentation are precisely “taught” during instrument setup. Misalignment of selected fiducials during the teaching process can shift measurement regions, and for small features, this can result in partially missing the targeted areas.

Targeting only the cell-containing regions of the slide not only has the potential to dramatically increase throughput but also reduce the amount of chemical noise collected during image acquisition. An overlay of the mass spectra from the cell-targeted approach (blue) and rectangular measurement region (red) is shown in Figure 3G. There is a noticeable decrease in the matrix-associated peaks for the targeted experiment and an increase in signal-to-noise for biologically relevant ions for the mean spectrum, especially in the low-mass region. The ability to annotate and target dispersed single cells is critical for improving throughput and minimizing “wasted” pixels (i.e., matrix-only regions) for single-cell MALDI imaging experiments.

## Conclusion

This study introduces a streamlined workflow for targeted MALDI IMS experiments, leveraging recently updated instrument control software and open-source tools for microscopy-driven segmentation. As high spatial resolution MALDI IMS becomes increasingly feasible, challenges such as longer acquisition times, data complexity, and increased resource demands have become significant barriers. Unlike current state-of-the-art approaches for multimodal studies, which often rely on custom scripts and in-house solutions, our workflow integrates seamlessly with existing software using the common open GeoJSON file format. The case studies presented—ranging from targeted cell types and tissue features in cerebral vasculature to kidney functional tissue units and dispersed single cells—demonstrate the method’s adaptability across various biological contexts. Moreover, these case studies employed diverse segmentation techniques, from simple manual annotations to automatically generated segments via machine learning, underscoring the workflow’s versatility. As MALDI IMS continues to advance in spatial resolution and scalability, targeted sampling will become more prevalent in biomedical studies, where large sample numbers are crucial to generalize findings across highly variable biological systems, particularly in human research. The method described herein offers the IMS community a vital tool to overcome these challenges, enabling more sophisticated experimental designs that address specific biological questions.

## Supporting Information Available

Annotation workflow, extended methods, additional instrument parameters, and whole slide images from the brain, kidney, and dispersed cell experiments (PDF)
