# Supplementary material for: A Streamlined Workflow for Microscopy-Driven MALDI Imaging Mass Spectrometry Data Collection
Source: J Am Soc Mass Spectrom. 2024 Nov 7;35(12):2795–800. doi: 10.1021/jasms.4c00365 (PMC11622233; doi:10.1021/jasms.4c00365)
Supplement: Supplementary file 1 — js4c00365_si_001.pdf [file js4c00365_si_001.pdf]

SUPPLEMENTAL INFORMATION

A Streamlined Workflow for Microscopy-Driven MALDI Imaging Mass Spectrometry Data Collection

Allison B. Esselman[1,2], Megan S. Ward[1,3], Cody R. Marshall[1,3], Ellie L. Pingry[1,4], Martin Dufresne[1,4], Melissa A. Farrow[1,4], Matthew Schrag[6,7,8], Jeffrey M. Spraggins[1,2,4,5,9]*

[1]Mass Spectrometry Research Center, Vanderbilt University, Nashville, TN 37240
[2]Department of Chemistry, Vanderbilt University, Nashville, TN 37240
[3]Chemical and Physical Biology Program, Vanderbilt University School of Medicine, Nashville, TN 37232
[4]Department of Cell and Developmental Biology, Vanderbilt University, Nashville, TN 37232
[5]Department of Biochemistry, Vanderbilt University, Nashville, TN 37232
[6]Department of Neurology, Vanderbilt University School of Medicine, Nashville, TN 37232
[7]The Vanderbilt Brain Institute, Vanderbilt University, Nashville, TN 37240
[8]Vanderbilt Memory and Alzheimer's Center, Vanderbilt University Medical Center, Nashville, TN 37232
[9]Department of Pathology, Microbiology, and Immunology, Vanderbilt University Medical Center, Nashville, TN 37232

*Jeffrey M. Spraggins; 465 21st Ave S. Room 9160, Medical Research Building III Vanderbilt University, Nashville, TN 37240; Fax: 615-343-8372; Phone: 615-343-9207; jeff.spraggins@vanderbilt.edu

Table of Contents

Figure S1	Annotation Workflow	S-2
Method S1	Extended Methods	S-2 – S-4
Table S1	Cell Detection Parameters in QuPath	S-5
Table S2	timsTOF fleX Instrument Parameters - Brain	S-5
Table S3	timsTOF fleX Instrument Parameters - Cells	S-6
Table S4	timsTOF fleX Instrument Parameters - Kidney	S-6
Figure S2	Whole Slide Images – Brain Experiment	S-7
Figure S3	Whole Slide Images – Kidney Experiment	S-8
Figure S4	Whole Slide Images – HeLa Cell Experiment	S-9

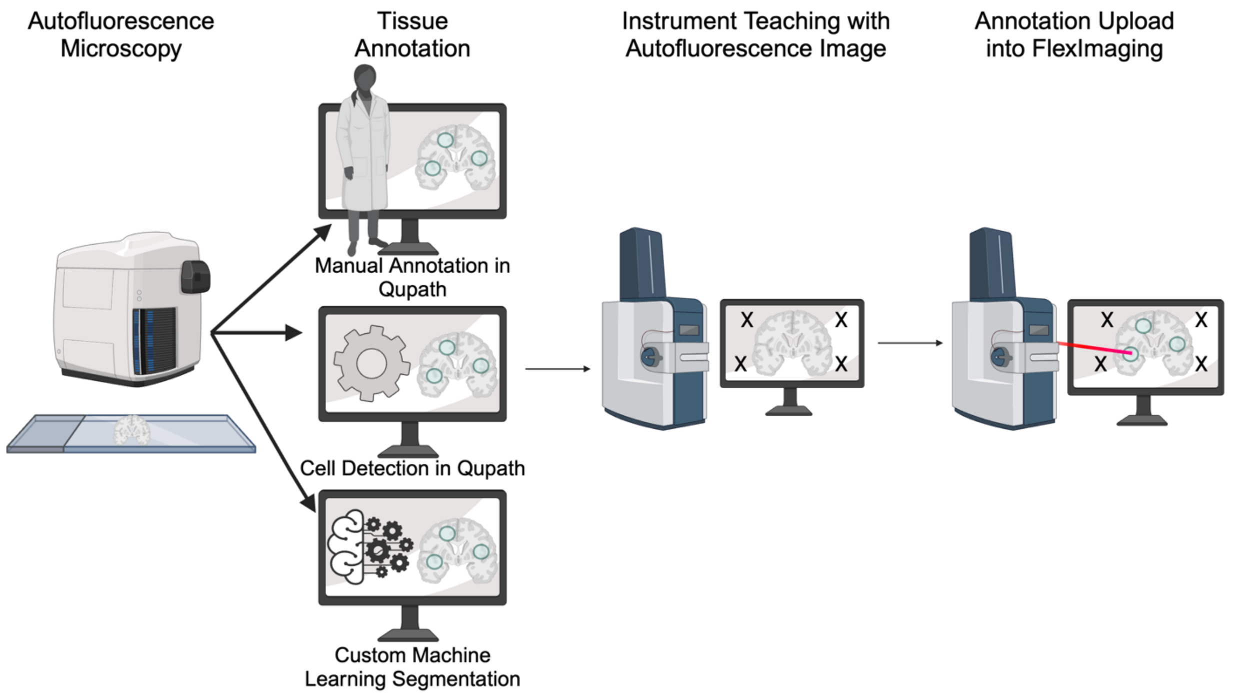

Figure S1. Annotation workflow for human brain (manual annotations in QuPath), HeLa cells (Cell Detection in QuPath), and human kidney (custom machine-learning segmentation model). The autofluorescence image used to create annotated regions was used to teach the instrument with flexImaging, and then the *.geojson* file of the regions was imported into flexImaging as ROIs.

<u>Methods S1</u>

Materials. Ammonium formate, carboxymethylcellulose sodium (CMC), glycerol, 10% neutral buffered formalin (NBF), Dulbecco's Phosphate Buffered Saline (DPBS modified, without calcium chloride and magnesium chloride), tris-buffered saline (TBS), fish gelatin, 100 mM glycine, 0.1% Triton X-100, bovine serum albumin, and 0.05% Tween-20 were purchased from Sigma-Aldrich (St. Louis, MO). The matrix 4-(dimethylamino)cinnamic acid (DMACA) with 99% purity, 20 mM Hoechst 33342, 4% paraformaldehyde (PFA), and Dulbecco's Modified Eagle Medium (DMEM with 4.5 g/L D-Glucose and L-Glutamine and without sodium pyruvate) were bought from Thermo Scientific (Waltham, MA). Fetal Bovine Serum (FBS) was purchased from Atlanta Biologicals (Flowery Branch, GA). High-performance liquid chromatography (HPLC)-grade acetone was purchased from Fisher Scientific (Pittsburgh, PA). Universal blocking reagent (10x) was purchased from BioGenex Laboratories (Fremont, CA). Antibody diluent reagent solution, 10X PBS Buffer (pH 7.4), and Ambion Nuclease Free Water were purchased from Invitrogen (Waltham, MA). Podocalyxin (AB208254), aquaporin 1 (AQP1) (AB225225), alpha smooth muscle actin (aSMA) (AB124964) and normal donkey serum (AB7475) were purchased from Abcam (Cambridge, UK). Collagen IV (M3F7) was purchased from NeoBiotechnologies (Union City, CA). Thiazine red was purchased from Chemsavers (Bluefield, VA). DAPI fluoromount-G was purchased from Southern Biotech (Homewood, Alabama). HeLa cells were purchased from American Type Culture Collection (Manassas, VA). Human kidney samples were provided by the Cooperative Human Tissue Network at Vanderbilt University Medical Center. Human brain samples were provided by the brain bank in Dr. Schrag's laboratory at Vanderbilt University.

Sample Preparation. The frontal cortex of a human brain donor with severe Alzheimer's disease and severe cerebral amyloid angiopathy was frozen in liquid nitrogen, embedded with 15% fish gelatin, and then stored in a -80 °C freezer. A normal portion of a kidney cancer nephrectomy was embedded in carboxymethylcellulose (CMC), flash-frozen in a bath of isopentane and dry ice, and stored in a -80 °C freezer.[1] Tissue was cryosectioned at 10 µm thickness using a CM3050 S cryostat (Leica Biosystems, Wetzlar, Germany). The sections were thaw-mounted onto indium tin oxide (ITO) coated glass slides (Delta Technologies, Loveland, CO). HeLa cells were cultured at 37°C and 5% CO_2 in DMEM with 10% FBS. Cells were seeded at 1.7 x 10^5 cells into a 10-cm plate containing an ITO slide. Cells were allowed to proliferate for approximately 48 hours. The HeLa cells were washed three times with DBPS and three times with chilled (4 °C) 150 mM ammonium formate for 45 seconds each. Slides were dried using nitrogen gas and a desiccator before storage in a -80 °C freezer.

Autofluorescence images of the samples were collected before IMS sample preparation with standard DAPI, eGFP, and DSRed fluorescent filters for the tissue samples and an additional brightfield channel for the cells using a Zeiss AxioScan.Z1 slide scanner (Carl Zeiss Microscopy GmbH, Oberkochen, Germany), equipped with a Colibri7 LED light source. To remove endogenous salts, tissue samples were then washed with chilled (4 °C) 150 mM ammonium formate 3 times for 45 seconds each and then dried with nitrogen gas to remove excess solution. (HeLa cells were washed before autofluorescence acquisition.) An in-house developed sublimation device was used to sublimate 5 mg of 4-(dimethylamino)cinnamic acid (DMACA) for kidney and dispersed cell samples and 12 mg for the brain sample onto the slide while heating (up to 175 °C) for 15 minutes under vacuum and cooling the sample to -78 °C using a dry ice and acetone slurry.[2] Following sublimation, the matrix was annealed by placing the slide onto a 100 °C hot plate for 15-20 seconds. After MALDI IMS data acquisition, post-IMS autofluorescence images were acquired using a Zeiss AxioScan.Z1 fluorescence slide scanner that uses an eGFP fluorescence filter and a monochromatic brightfield image.

Stain Microscopy. After the collection of the post-IMS autofluorescence image, stain microscopy was performed. For the brain tissue, the matrix was removed with a series of ethanol (EtOH) washes (70% to 90% EtOH). The tissue was then fixed with a 4% PFA solution for 15 minutes. The PFA was removed with a series of increasing sugar solutions (10% to 30% sucrose). The tissue was then placed in a sealed petri dish with 1X TBS and photobleached for 48 hours with an LED lamp at 4°C (BESTVA DC Series 1200W LED Grow Light Full Spectrum). Sections were then washed with 100 mM glycine/1X TBS/0.1% Triton X-100 buffer for 30 minutes. Sections were blocked for 60 minutes at 37°C in a solution containing 10% normal donkey serum in 1X TBS/0.1% bovine serum albumin. Primary antibodies for Collagen IV, aSMA, and thiazine red were used to stain the tissue. Tissue was washed 4 times for 10 minutes each in 1X TBS/0.05% Tween-20 then coverslipped with a DAPI fluoromount.

The kidney tissue section was fixed with 10% NBF by submerging the tissue for 5 minutes. The tissue sections were then washed with 1X PBS, blocked for 30 minutes with 1X universal blocking reagent, and incubated with podocalyxin and AQP1 for 2 hours at room temperature. The tissue section was then washed with 1X PBS and incubated with Hoechst 33342 for 10 minutes before attaching a coverslip using a 50:50 glycerol:H_2O solution. Immunofluorescence stains were

imaged using a Zeiss AxioScan.Z1 fluorescence slide scanner using standard AF488, AF594, AF647, and DAPI filters.

Following post-IMS autofluorescence imaging, HeLa cells were fixed with 10% NBF for 6 minutes. Cells were then washed three times with 1X PBS and stained with Hoechst 33342 for 10 mins. After staining, cells were washed three times with 1X PBS and coverslipped with a 50:50 glycerol:H_2O solution. The nuclear stain was imaged via a Zeiss AxioScan.Z1 fluorescence slide scanner using H3342 and brightfield.

Tissue Feature Annotations. All segmentation was performed on the sample's autofluorescence image. Manual vasculature segmentation of the human brain was achieved with the wand and brush tools in QuPath, and dispersed cell segmentation was performed with the Cell Detection feature in QuPath. The specific parameters for this detection can be found in **Table S1.** Segmentation of glomeruli in human kidney was performed using a previously described method.[3–5] The FTU segmented areas were scaled by a factor of 1.4 using an affine scale transform to allow for slight errors in spatial targeting and to ensure the capture of the border of each glomerulus.

MALDI IMS. The experiments were performed on a timsTOF fleX mass spectrometer with a microGRID stage (Bruker Daltonics, Bremen, Germany) in negative ionization qTOF mode. The autofluorescence image of each sample was used as the teaching image in flexImaging. Once the instrument was taught, the segmentations were imported into flexImaging using the new import regions feature to define measurement regions so that only the glomeruli, vasculature, and dispersed cells were measured by MALDI IMS.

Human brain vasculature imaging data from 73,181 pixels and 17 regions were acquired at 5 µm IMS pixel size using 25 shots per pixel and 5.4% total laser power averaging ~12 pixels/second from *m/z* 500 to 2600. Instrument-specific parameters are available in **Table S2**.

Dispersed HeLa cell imaging data from 53,517 pixels and 999 QuPath cell detection annotations and a rectangular control region of 642,747 pixels was acquired. Both experiments were acquired at 5 µm IMS pixel size using 25 shots per pixel and 4.8% total laser power from m/z 450 to 1600. **Table S3** details instrument-specific parameters.

Human kidney tissue imaging data from 725,238 pixels and 305 regions were collected at 5 µm IMS pixel size using 25 shots per pixel and 4.5% total laser power averaging ~12 pixels/second from *m/z* 300 to 2000. Instrument-specific parameters are available in **Table S4.**

Table S1. QuPath Cell Detection Parameters for Automated Segmentation.

Setup Parameters	
Requested Pixel Size	0.5 µm
Nucleus Parameters	
Background Radius	10 µm
Mean Filter Radius	0 µm
Sigma	1.5 µm
Minimum Area	10 μm^2
Maximum Area	3000 μm^2
Intensity Parameters	
Threshold	15
Cell Parameters	
Cell Expansion	20
General Parameters	
Smooth Boundaries	Yes
Make Measurements	Yes

Table S2. Instrument Parameters for the Human Brain Experiment.

**Negative Ion Mode**	
Transfer	
MALDI Plate Offset	70.0 V
Deflection 1 Delta	-70.0 V
Funnel 1 RF	400.0 Vpp
isCID Energy	0.0 eV Vpp
Funnel 2 RF	400.0 Vpp
Multipole RF	500.0 Vpp
Collision Cell	
Collision Energy	10.0 eV
Collision RF	3000.0 Vpp
Quadrupole	
Ion Energy	10.0 eV
Low Mass	500.00 *m/z*
Focus Pre TOF	
Transfer Time	120.0 µs
Pre Pulse Storage	12.0 µs

Table S3. Instrument Parameters for the Dispersed HeLa Cell Experiment.

Negative Ion Mode	
Transfer	
MALDI Plate Offset	70.0 V
Deflection 1 Delta	-70.0 V
Funnel 1 RF	350.0 Vpp
isCID Energy	5.0 eV Vpp
Funnel 2 RF	300.0 Vpp
Multipole RF	300.0 Vpp
Collision Cell	
Collision Energy	10.0 eV
Collision RF	2000.0 Vpp
Quadrupole	
Ion Energy	5.0 eV
Low Mass	*m/z* 400.00
Focus Pre TOF	
Transfer Time	80.0 µs
Pre Pulse Storage	8.0 µs

Table S4. Instrument Parameters for the Human Kidney Experiment.

Negative Ion Mode	
Transfer	
MALDI Plate Offset	60.0 V
Deflection 1 Delta	-70.0 V
Funnel 1 RF	350.0 Vpp
isCID Energy	10.0 eV Vpp
Funnel 2 RF	400.0 Vpp
Multipole RF	500.0 Vpp
Collision Cell	
Collision Energy	10.0 eV
Collision RF	2500.0 Vpp
Quadrupole	
Ion Energy	5 eV
Low Mass	*m/z* 550.00
Focus Pre TOF	
Transfer Time	90 µs
Pre Pulse Storage	12.0 µs

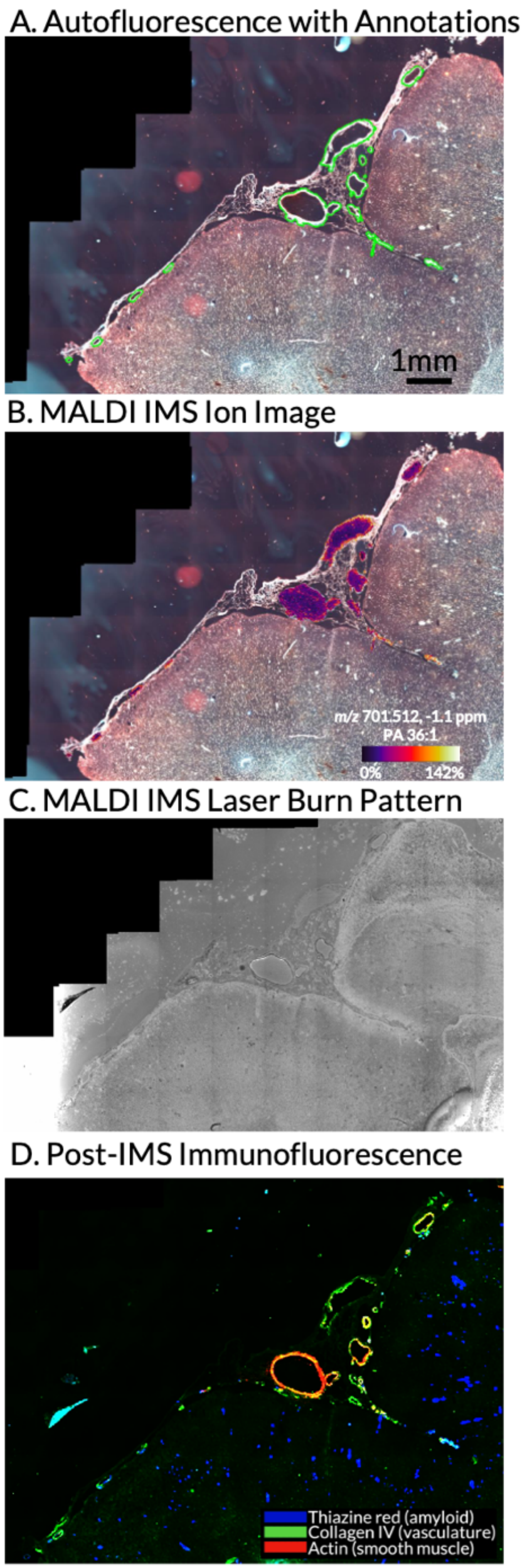

Figure S2. Whole slide images from the targeted multimodal imaging of vasculature in the frontal cortex of the human brain. The autofluorescence image of the tissue section was used to manually annotate 17 vasculature ROIs in the tissue section (A). A MALDI IMS ion image of vasculature ROIs overlayed onto the autofluorescence image demonstrates the heterogeneity of PA (36:1) (*m/z* 701.512, -1.1 ppm) within the vasculature regions (B). The post-MALDI IMS brightfield microscopy of the laser ablation craters (C), and post-MALDI IMS IF stain show the specific targeting of the vasculature with this MALDI IMS experiment (D). In the IF image, amyloid is shown in blue (thiazine red), vasculature is shown in green (collagen IV), and smooth muscle is shown in red (actin).

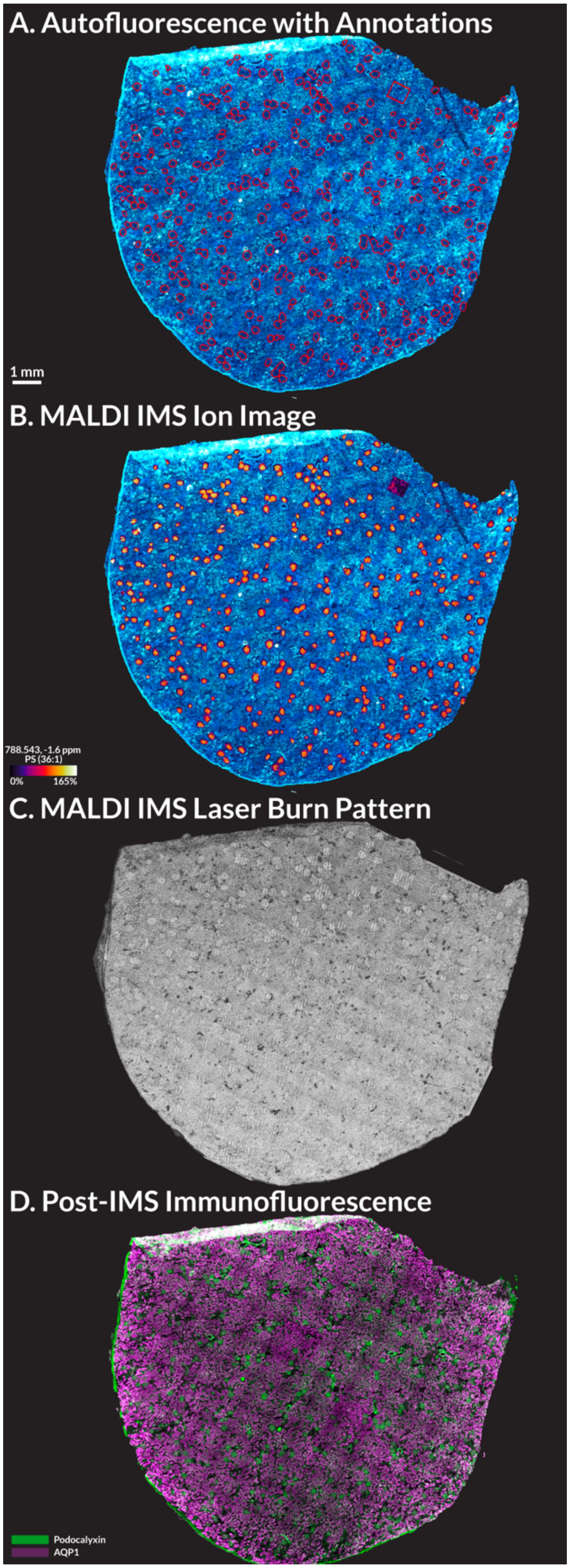

Figure S3. Whole slide images from the specific targeting of glomeruli in human kidney. The autofluorescence image of the tissue section was used with a custom segmentation model to annotate 304 glomeruli in the tissue section (A). A MALDI IMS ion image of glomeruli overlayed onto the autofluorescence image shows the localization of PS (36:1) (*m/z* 788.543, -1.6 ppm) specifically to glomeruli (B). The post-MALDI IMS acquisition laser burn pattern (C), and post-MALDI IMS IF stain show the specific targeting of glomeruli in the tissue section. In the IF image, glomeruli are shown in green (podocalyxin), and proximal tubules are shown in purple (aquaporin 1) (D).

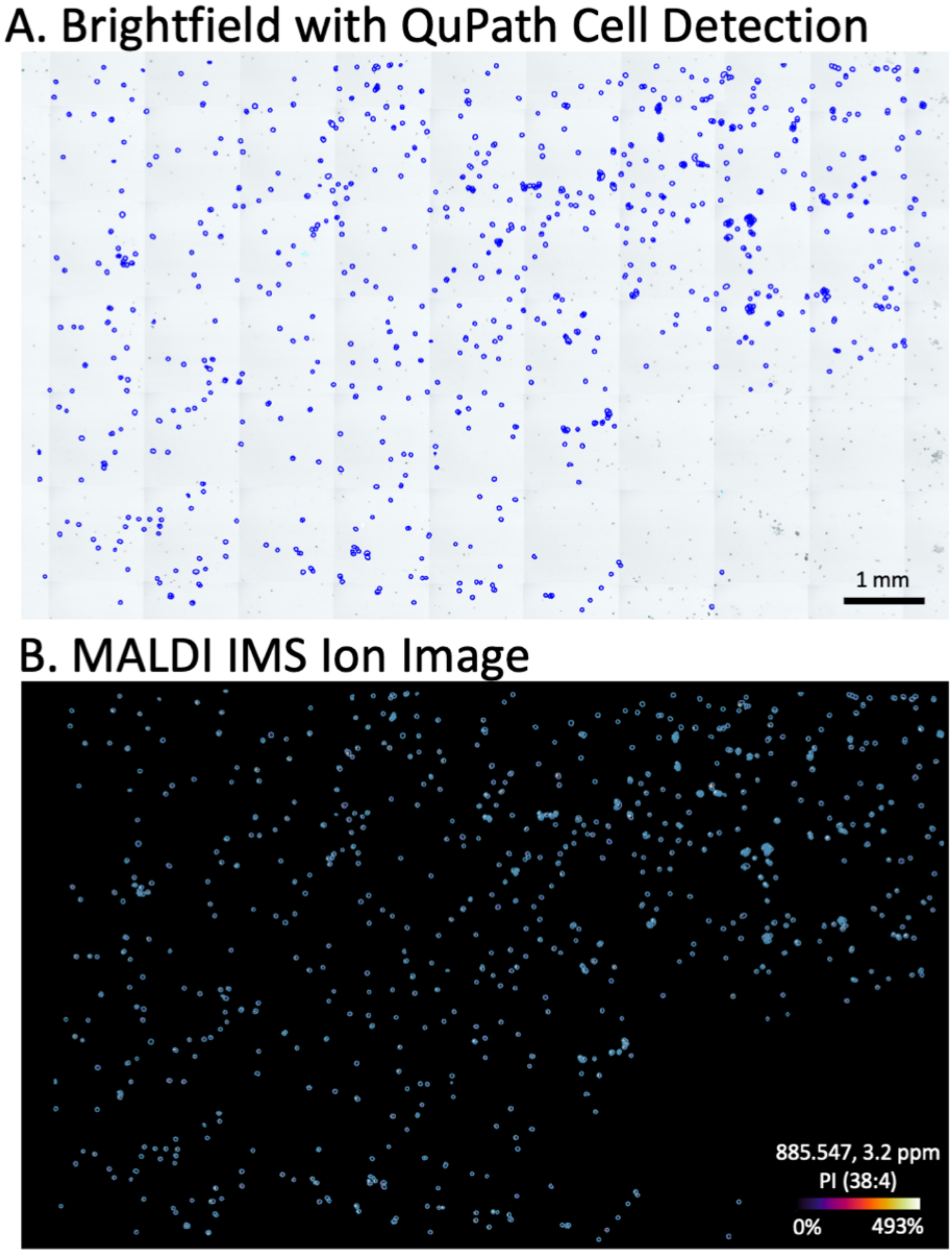

Figure S4. Targeted acquisition of HeLa cells. The QuPath Cell Detection feature was used on a brightfield image to define 999 ROIs (A). A MALDI IMS ion image of PI (38:4) (*m/z* 885.547, 3.2 ppm) localized to only the HeLa cells (B).
